# Supplementary material for: Content-rich biological network constructed by mining PubMed abstracts
Source: BMC Bioinformatics. 2004 Oct 8;5:147. doi: 10.1186/1471-2105-5-147 (PMC528731; doi:10.1186/1471-2105-5-147)
Supplement: Additional File 5 — The original Chilibot query results of the term "long-term potentiation (LTP)" and 22 other terms, limiting the latest references analyzed to the years 1990, 1995, 2000, and 2004. [file 1471-2105-5-147-S5.bz2 › chilibotAdditionalFile5/ltp2000/html/SYNAPSIN I_SYNAPTOPHYSIN.html]

 


 **SYNAPSIN I** and **SYNAPTOPHYSIN** 
  
Found 57 abstracts in PubMed,  **30 abstracts were retrieved and analyzed**.  


---

 Search Google  |
 PDF files only 
|  EDU domain only 

---

**Interactive relationship** (e.g. stimulation, inhibition, etc)

- After training, pp60c src from hippocampus showed enhanced interactions with synaptic proteins such as  **synapsin I** ,  **synaptophysin** , and the type 2 N methyl d aspartate receptor, as well as the cytoskeletal protein actin.  Ref: 10884433 Proc Natl Acad Sci U S A, 2000

**Parallel relationship** (e.g. studied together, co-existance, homology, etc.)

- Horseradish peroxidase filled vacuoles as wells as vesicles contained the synaptic vesicle membrane proteins VAMP II, synaptotagmin, SV2, and  **synaptophysin** , the vesicle associated proteins rab 3A and  **synapsin I** , and in addition SNAP 25.  Ref: 10579591 Neuroscience, 1999
- No synaptotagmin I like immunoreactivity was found in large axons, while accumulations of the synaptic vesicle proteins  **synaptophysin**  and  **synapsin I**  were found in all types of axons.  Ref: 7534885 Neuroscience, 1994
- Decreased immunoreactivity for  **synaptophysin** ,  **synapsin I** , SNAP 25, and syntaxin I was observed in the cerebral cortex in every case, thus showing generalized reduction of proteins involved in exocytosis of synaptic vesicles in the brains of patients with spongiform encephalopathy.  Ref: 10343324 Neurobiol Dis, 1999
- A decrease in the synaptic protein  **synapsin I** ,  **synaptophysin**  content was detected at 10 8 M of TMT in synaptosomal fractions.  Ref: 7496810 Brain Res, 1995
- Second, using this system, we examined localization of  **synapsin I**  and  **synaptophysin**  in nerve endings after electrical stimulation.  Ref: 7820876 Cell Struct Funct, 1994
- Redistribution of  **synapsin I**  and  **synaptophysin**  in response to electrical stimulation in the rat neurohypophysial nerve endings.  Ref: 7820876 Cell Struct Funct, 1994
- In order to characterize synaptic involvement in human spongiform encephalopathies, the expression of synaptic vesicle associated proteins,  **synaptophysin**  and  **synapsin I** , and presynaptic plasma membrane proteins, synaptosomal associated protein of 25 kDa SNAP 25 and syntaxin I, was examined in the brains of four patients who had suffered from sporadic Creutzfeldt Jakob disease.  Ref: 10343324 Neurobiol Dis, 1999
- Other proteins including the synaptic vesicle proteins  **synapsin I**  and  **synaptophysin**  remained unaltered.  Ref: 7605916 Neuroreport, 1995
- Similar expression profiles for the two proteins were also observed in immunocytochemical studies of Xenopus spinal neurons in culture intense staining of  **synaptophysin**  was found on the first day, while  **synapsin I**  was not detected until after three days in culture.  Ref: 8895877 Neuroscience, 1996
- We conclude that  **synapsin I**  is a reliable, sensitive immunohistochemical marker for neuronal neuroendocrine differentiation in human neoplasms and may offer some advantages over  **synaptophysin**  when applied to formalin fixed, paraffin embedded tissues, particularly in the evaluation of primitive neuroectodermal tumors and neuroendocrine tumors.  Ref: 8287627 Clin NeuropatholClin Neuropathol, 1996
- Rab3a immunoreactivity was always colocalized with  **synaptophysin**  IR and  **synapsin I**  IR in nerve terminals of the spinal cord and peripheral nerve endings.  Ref: 8720497 Brain Res, 1996
- In the outer plexiform layer OPL,  **synaptophysin**  appeared at P4, while  **synapsin I**  and syntaxin appeared at P8.  Ref: 9352109 Brain Res Dev Brain Res, 1997
- Furthermore, expression of  **synaptophysin** ,  **synapsin I** , syntaxin I and SNAP 25 is the same in the cerebellum of irradiated and normal rats from this time to adulthood 3 months.  Ref: 10479063 Int J Dev Neurosci, 1999
- Accumulation of Rab3a was accompanied by accumulation of  **synaptophysin**  and  **synapsin I** , two synaptic vesicle membrane proteins, and accumulation of clathrin light chain.  Ref: 8521869 Eur J Cell Biol, 1995
- VAChT containing varicosities in the Renshaw cell area, with a probable origin from motoneuron axon collaterals, exhibited CSP,  **synapsin I**  II, and synaptobrevin 1 LI... syntaxin, synuclein,  **synapsin I** , synaptotagmin I, synaptotagmin I II,  **synaptophysin**  and synaptobrevin 2 LI.  Ref: 10421869 J Comp Neurol, 1999
- The presence of synaptic vesicle associated proteins such as  **synaptophysin** , synaptotagmin and  **synapsin I**  in the growth cones of regenerating axons indicates the possibility that exocytotic fusion of vesicles with the surface axolemma supplies the membranous components for the extension of regenerating axons.  Ref: 8829147 Neurosci Res, 1996
- The distribution of four proteins associated with synaptic vesicles, SV2,  **synaptophysin** ,  **synapsin I** , and rab3a, was investigated during postnatal development of the posteromedial barrel subfield in the rat somatosensory cortex.  Ref: 8915833 J Comp Neurol, 1996
- Immunohistochemistry in combination with confocal laser microscopy showed the presence of synaptosomal associated protein of 25 kDa, syntaxin, cysteine string protein, synuclein,  **synapsin I** ,  **synapsin I**  II, synaptotagmin I, synaptotagmin I II,  **synaptophysin** , and synaptobrevin 2 like immunoreactivity in VAChT containing C terminals.  Ref: 10421869 J Comp Neurol, 1999
- Immunohistochemistry of  **synapsin I**  and  **synaptophysin**  in human nervous system and neuroendocrine tumors.  Ref: 8287627 Clin NeuropatholClin Neuropathol, 1999
- To understand the dynamics of synaptic vesicles and  **synapsin I** , we have studied the localization of  **synapsin I**  and  **synaptophysin**  in resting and stimulated nerve endings by ultracryomicrotomy and colloidal gold immunocytochemistry.  Ref: 7820876 Cell Struct Funct, 1994
- Expression of  **synaptophysin** ,  **synapsin I**  and syntaxin was studied immunocytochemically in the developing rat retina using indirect immunoperoxidase technique.  Ref: 9352109 Brain Res Dev Brain Res, 1997
- In crushed sciatic nerve axons, Rab3a was colocalized, proximal to the crush, with  **synaptophysin**  IR,  **synapsin I**  IR, CGRP IR, and TH IR, but only partially co localized with NPY IR and SP IR.  Ref: 8720497 Brain Res, 1996
- The competence of axons to form presynaptic vesicle clusters in response to contact with the somata or dendrites of mature or immature neurons was determined by immunofluorescent staining for  **synapsin I**  or  **synaptophysin** .  Ref: 7965070 J Neurosci, 1994
- Intense immunoreactivity for both  **synapsin I**  and  **synaptophysin**  was observed in tumors containing well differentiated neurons gangliocytoma, ganglioglioma, neurocytoma.  Ref: 8287627 Clin NeuropatholClin Neuropathol, 1994
- Determination of  **synapsin I**  and  **synaptophysin**  in body fluids by two site enzyme linked immunosorbent assays.  Ref: 9692851 J Immunol Methods, 1998
- High magnification revealed that synaptotagmin I like immunoreactivity was mainly distributed in a fine granular pattern, but large, brightly fluorescent granules which were not labelled by anti  **synaptophysin**  or anti  **synapsin I**  were occasionally observed.  Ref: 7534885 Neuroscience, 1994
- Two site enzyme linked immunosorbent assays ELISA have been established for the specific and sensitive determination of two membrane proteins of the small synaptic vesicles SSV, namely peripheral  **synapsin I**  and integral  **synaptophysin** .  Ref: 9692851 J Immunol Methods, 1998
- In the inner plexiform layer IPL, syntaxin immunoreactivity appeared at postnatal day 1 P1 whereas  **synaptophysin**  and  **synapsin I**  staining were first observed at P2.  Ref: 9352109 Brain Res Dev Brain Res, 1997
- Some PNETs and neuroendocrine tumors that were immunoreactive for  **synapsin I**  did not stain for  **synaptophysin** .  Ref: 8287627 Clin NeuropatholClin Neuropathol, 1997
- Concomitantly, the two synaptic vesicle proteins dropped,  **synaptophysin**  > 50% and  **synapsin I**  > 85%.  Ref: 7783952 Neurosci Lett, 1995
- In the present study, immunohistochemistry to the presynaptic membrane proteins syntaxin I and synaptosomal associated protein of 25 kDa SNAP 25 synaptic vesicle associated proteins  **synaptophysin**  and  **synapsin I**  and the neuronal maturation and axonal growth related protein GAP 43, has been carried out in the normal developing cerebellum and following a single dose of ionizing radiation 2 Gy gamma rays at postnatal day 1.  Ref: 10479063 Int J Dev Neurosci, 1999
- Developmental expression of  **synaptophysin** ,  **synapsin I**  and syntaxin in the rat retina.  Ref: 9352109 Brain Res Dev Brain Res, 1997
- In parallel, neurotoxic effects were assessed by determining the content of  **synaptophysin**  and  **synapsin I** , both in the total homogenates and in the synaptosomal fraction of the cultures.  Ref: 7496810 Brain Res, 1995
- **Synapsin I**  was purified by conventional means from human and porcine brain and  **synaptophysin**  was purified by immunoaffinity chromatography from porcine brain.  Ref: 9692851 J Immunol Methods, 1998
- Furthermore, these vesicle clusters stained positively for two SV associated proteins,  **synapsin I**  and  **synaptophysin** , by EM immunocytochemistry ICC.  Ref: 8583500 J Neurosci Res, 1995
- the levels of  **synaptophysin**  p38,  **synapsin I**  and sv2... correlate with the extent of FM1 43 labelling at synapses.  Ref: 9153578 Eur J Neurosci, 1997
- SV2,  **synapsin I** , and  **synaptophysin**  revealed the organization of the barrel field in the neonate.  Ref: 8915833 J Comp Neurol, 1996
- Indirect immunofluorescence with Texas Red as label was used to investigate the distribution of 3 different groups of immunogens enzymes monoamine oxidase A and B, receptors beta adrenoceptor protein, and synaptic vesicle proteins  **synapsin I**  and  **synaptophysin**  in each cortical slice.  Ref: 7967715 J Neurosci Methods, 1994
- In contrast, the protein was found in muscle spindles of young rats and in perivascular terminals, where it co localized with  **synaptophysin**  and  **synapsin I** .  Ref: 7534885 Neuroscience, 1994
- After 5 days of treatment, cultures were analyzed for the presence of synapses by  **synapsin I**  and  **synaptophysin**  antibody labeling and by electron microscopy.  Ref: 7568106 Proc Natl Acad Sci U S A, 1995
- Parallel increases in  **synaptophysin**  immunoreactivity were not observed, thus suggesting a selective induction of  **synapsin I**  after ischemia.  Ref: 10196445 Brain Res, 1999
- Of these latter proteins, one is integral  **synaptophysin**  the other peripheral  **synapsin I**  to the synaptic vesicle membranes.  Ref: 7783952 Neurosci Lett, 1995
- In most cases, immunostaining for  **synapsin I**  was sharper and often more intense than for  **synaptophysin** .  Ref: 8287627 Clin NeuropatholClin Neuropathol, 1995
- Using the ELISA, neither  **synapsin I**  nor  **synaptophysin**  could be determined in serum or cerebrospinal fluid CSF from healthy donors or patients suffering various neurological disorders or pheochromocytomas.  Ref: 9692851 J Immunol Methods, 1998
